# Evaluation of in-vivo antidiarrhoeal and in-vitro antibacterial activities of the root extract of *Brucea antidysenterica* J. F. Mill (Simaroubaceae)

**DOI:** 10.1186/s12906-020-03001-7

**Published:** 2020-06-30

**Authors:** Kaleab Alemayehu Zewdie, Dayananda Bhoumik, Dawit Zewdu Wondafrash, Kald Beshir Tuem

**Affiliations:** Department of Pharmacology and Toxicology, School of Pharmacy, Mekelle University, 1871 Mekelle, Ethiopia

**Keywords:** Antidiarrhoeal, Antibacterial, Castor oil, Agar well diffusion, *Brucea antidysenterica*

## Abstract

**Background:**

Diarrhoea has been the major cause of death especially in children of developing countries. *Brucea antidysenterica* is one of the several medicinal plants used traditionally for the treatment of diarrhoea in Ethiopia. Hence, the present study was undertaken to investigate the antidiarrhoeal and antibacterial activities of the root extract of *B. antidysenterica.*

**Methods:**

Plant material was extracted by maceration technique using 80% methanol. The antidiarrhoeal activity was tested using castor oil-induced diarrhoea, castor oil-induced charcoal meal test, and castor oil-induced enteropooling models in mice. Whilst, the antibacterial activity of the crude extract was evaluated using agar well diffusion and broth microdilution methods.

**Results:**

The 80% methanolic crude extract significantly delayed the diarrhoeal onset at the two higher doses (*p* < 0.001) and it has also inhibited the number and weight of faecal output at all tested doses as compared with the negative control. Moreover, it showed a significant anti-motility effect (*p* < 0.001) at all tested doses. Whereas it displayed a significant reduction in the weight and volume of intestinal contents at the doses of 200 and 400 mg/kg (*p* < 0.01). The highest concentration (800 mg/mL) of test extract showed maximum zone of inhibition in all tested standard strains of bacteria (18.3 mm–22 mm). While MIC and MBC values (0.39 mg/mL and 1.56 mg/mL) showed that *S. flexneri* was the most susceptible pathogen for test extract.

**Conclusion:**

The study revealed that the root extract of *B. antidysenterica* has antidiarrhoeal and antibacterial activities.

## Background

Diarrhoea is a loss of watery stool at least three times a day [1], or more frequent bowel movements with a volume or weight of greater than 200 mL or 200 g in 24 h duration [2, 3]. Although diarrhoea is a preventable condition, it affects almost all global population and accountable for 5% of health defects and 4% of all deaths worldwide [4]. Globally, about 2.2 million people died each year and most are due to bacterial-induced diarrhoea. Several bacterial pathogens including *Vibrio cholera*, *Clostridium difficile*, *Shigella* species, *Escherichia coli*, *Pseudomonas aeruginosa* and *Salmonella* species causes various forms of diarrhoea [5, 6]. According to the Centre for Disease Control and Prevention (CDC) and WHO report in 2017, 1 in 9 child death was reported worldwide, which makes diarrhoea the second leading cause of death for under-five children [1, 5]. Moreover, diarrhoea kills 2195 children each day which is greater than malaria, measles and AIDS combined [5, 7].

Since primaeval times, humans used natural products to relieve and treat diseases for themselves and their livestock. Different traditional plants have been used as a source of modern medicine [8, 9]. In addition, numerous traditional plants were scientifically reported for their antidiarrhoeal activity:- including *Myrtus communis* [10]*, Lantana camara* [11]*, Croton macrostachyus* [12]*, Carissa carandas* [13]*, Zehneria scabra* [14]*, Ajuga remota* [15]*, Discopodium penninervum* [16] and *Lepidium sativum* [17].

*Brucea antidysenterica* J. F. Mill (Family: Simaroubaceae) belongs to genus *Brucea*. It is a small tree, widely distributed in tropical America and Africa [18]. The family Simaroubaceae comprises about 25 genera, 120 species and in the genus *Brucea* about 10 species are found in Africa, Asia, and Australia. *B. antidysenterica* is mostly found in Ethiopia and well-known for its medicinal uses [19, 20]. It has been reported for several ethnomedicinal uses such as malaria [20], bacterial infections [21–23], dysentery [24] and amoebicidal effect [25].

The anti-diarrhoeal claims of *B. antidysenterica* were stated in numerous works of literature [26–29]. The bark, fruit, and roots of *B. antidysenterica* have been used by traditional society against dysentery, as an anthelmintic and for treatment of fever. The seed, leaf and roots are used as a remedy for diarrhoea, indigestion, and stomach-ache [26, 29]. The present study was investigated to assess the antidiarrhoeal and antibacterial activities of the 80% hydro-methanolic root extract of *B. antidysenterica* in laboratory-based studies.

## Methods

### Drugs and chemicals

Castor oil (Amman Pharmaceutical industries CO, Jordan), activated charcoal (Research-Lab fine Chem industries, Mumbai, India), loperamide (Daehwa Pharmaceuticals, Republic of Korea), distilled water (Dallul Pharmaceuticals PLC, Addis Ababa, Ethiopia), methanol (Alpha Chemika, Mumbai, India), ciprofloxacin 5 μg/disc (Becton, Dickinson and Company, Sparks, USA), ceftazidime 30 μg/disc (Oxoid Ltd., Basingstoke, England), muller hinton agar (MHA) (HiMedia Laboratories Pvt. Ltd., India), muller hinton broth (MHB) (HiMedia Laboratories Pvt. Ltd., India) and nutrient agar (Micro master lab, India) were used during the study.

### List of materials

The following materials were used for the study. Electrical grinder (Shanghai Yuan Wo industrial and trade CO. LTD, Shanghai city), orbital shaker (Stuart S01, UK), drying oven (M200CFL, England), vortex mixer (Assistant Reamix 2789, Germany) and Bio-safety Cabinet (Bio- II- A/P, Telstar Company, Italy).

### Collection and authentication of plant material

The roots of *B. antidysenterica* were collected from one of the claimed areas ‘*Lumame*’, in Gozamin Wereda, east Gojjam zone of Amhara region, Ethiopia in November 2018. Identification and authentication of the plant material was done by Professor Sileshi Nemomissa, an authorized botanist at the National Herbarium, College of Natural and Computational Sciences, Addis Ababa University, and the sample were deposited for future reference with voucher specimen number 1.

### Preparation of crude extract

The collected root was washed gently and dried at room temperature under shade. It was then chopped manually and ground with an electrical grinder. The extraction was done by maceration technique with 80% methanol solvent, in the 1:6 solute-solvent ratio. The extraction was facilitated by intermediate manual shaking and occasional quivering with an orbital shaker at 120 rpm. After 72 h, the macerate was filtered with a double-layered muslin cloth and Whatman number 1 filter paper. The marc was re-macerated twice with fresh solvent for exhaustive extraction. The combined extract was dried under 40 °C in a drying oven. After drying, the percentage yield of plant extract was determined and it was 6.26% w/w. Finally, the dried crude extract was stored in the refrigerator at 4 °C until use [20].

### Experimental animals

Healthy Swiss albino mice of either sex (weighing 25–35 g and age of 6–8 weeks) were used for the experiment. The animals were obtained from and maintained in the laboratory of Department of Pharmacology and Toxicology, School of Pharmacy, Mekelle University. All animals were housed in plastic cages at room temperature in an air-conditioned room of 12-h light/dark cycle with accesses of pellet diet and clean water ad libitum. Before any experiment was started animals were allowed a week of acclimatization to the experimental environment and all the experiments were carried out according to the internationally accepted laboratory animal care and use guideline [30, 31]. The protocol approval letter was obtained from the Health Research Ethics Review Committee (HRERC) of College of Health Sciences, Mekelle University and registered as ERC 1537/2018 protocol number on December 11, 2018.

### Acute Oral toxicity test

Acute oral toxicity test was performed based on the Organization for Economic Cooperation and Development (OECD) guideline, number 425. All animals were observed continuously for toxicities like diarrhoea, decrease of appetite, hair erection and loss, lacrimation, convulsion, salivation, lethargy, paralysis, and mortality for the first 1 hour continuously and intermittently for the next 3 hours and periodically for 24 h and later cage side observation continued for 14 days [31].

### Grouping and dosing of animals

Mice were randomly assigned into five groups of six animals each to perform antidiarrhoeal activity using three models. The negative control groups were treated with distilled water (DW) (10 mL/kg), positive control groups received the standard drug loperamide (3 mg/kg) in all models. The three tested doses were selected based on the result of acute toxicity study. Based on that 100, 200 and 400 mg/kg were considered as low dose, middle dose and high dose, respectively. A volume of 1 mL/100 g was administered orally based on individual mouse body weight [31]. The duration of administration was depending on the type of test and it was described along with the respective models.

### Determination of anti-diarrhoeal activity

#### Castor oil induced Diarrhoea model

This model was conducted according to Degu et al., and Sisay et al., [10, 12]. Animals were fasted for 18 h and placed individually in a cage, in which the bottom floor was lined with blotting paper and was replaced every hour. Then animals received either vehicle or treatment samples as described in section 2.7 based on their fasting weight. Diarrhoea was induced by administering 0.5 mL of castor oil per oral route to each mouse just 1 h after the previous treatments. The observation was continued for a period of 4 h. Time of onset of diarrhoea, a total number of faecal outputs (frequency of defecation) and weight of faeces excreted by the animals were recorded. Finally, the percentage of diarrhoeal inhibition, percentage weight of wet faecal output and percentage weight of total faecal output were determined with respect to their formula [10, 12, 14].

$$ \%\mathrm{Inhibition}=\frac{\mathrm{Average}\ \mathrm{number}\ \mathrm{of}\ \mathrm{WFC}-\mathrm{Average}\ \mathrm{number}\ \mathrm{of}\ \mathrm{WFT}}{\mathrm{Average}\ \mathrm{number}\ \mathrm{of}\ \mathrm{WFC}}\times 100 $$

Where, WFC = average number of wet faeces in the control group and

WFT = average number of wet faeces in the test group.

$$ \%\mathrm{of}\ \mathrm{Wet}\ \mathrm{faecal}\ \mathrm{output}=\frac{\mathrm{Mean}\ \mathrm{weight}\ \mathrm{of}\ \mathrm{wet}\ \mathrm{faces}\ \mathrm{of}\ \mathrm{each}\ \mathrm{group}\ }{\mathrm{Mean}\ \mathrm{weight}\ \mathrm{of}\ \mathrm{wet}\ \mathrm{faces}\ \mathrm{of}\ \mathrm{the}\ \mathrm{control}}\times 100 $$

$$ \%\mathrm{of}\ \mathrm{Total}\ \mathrm{faecal}\ \mathrm{output}=\frac{\mathrm{Mean}\ \mathrm{faecal}\ \mathrm{weight}\ \mathrm{of}\ \mathrm{each}\ \mathrm{group}}{\mathrm{Mean}\ \mathrm{weight}\ \mathrm{of}\ \mathrm{the}\ \mathrm{control}}\times 100 $$

#### Castor oil induced charcoal meal test

Gastrointestinal motility test by using activated charcoal was done in accordance with Sisay et al., [10]. Mice of either sex were fasted for 18 h with free access to water and treated with the vehicle, standard drug and plant extract according to their respective group as described in section 2.7 based on their fasting weight by oral gavage. After 1 hour of test/ vehicle compound administration, 0.5 mL castor oil was administered by oral gavage then 1 mL of 5% charcoal suspension was administered orally 1 hour after castor oil treatment. After 1 hour of the charcoal meal, animals were sacrificed by cervical dislocation and the small intestine was dissected out and later the total length covered by a charcoal indicator from the pylorus to cecum was measured and calculated as a percentage of the total length of the small intestine. Finally, the Peristalsis index and proportion of inhibition were calculated by using the following formula [10, 12, 32].

$$ \mathrm{Peristaltic}\ \mathrm{index}=\frac{\mathrm{Mean}\ \mathrm{distance}\ \mathrm{travelled}\ \mathrm{by}\ \mathrm{charcoal}\ \mathrm{meal}\ }{\mathrm{Mean}\ \mathrm{length}\ \mathrm{of}\ \mathrm{small}\ \mathrm{intestine}}\times 100 $$

$$ \%\mathrm{of}\ \mathrm{inhibition}=\frac{\mathrm{PIC}-\mathrm{PIT}}{\mathrm{PIC}}\times 100 $$

Where, PIC = Peristaltic index of control; PIT = Peristaltic index of the test group

#### Castor oil induced Enteropooling model

The intraluminal fluid accumulation (enteropooling) was carried out based on a method described by Sisay et al., [10]. Mice were grouped as described earlier and fasted for 18 h prior to the experiment. Then the test compound and vehicle were given according to their grouping based on their fasting weight by oral gavage 1 hour prior to castor oil administration. After 1 hour all mice were sacrificed by cervical dislocation, and then the small intestine was isolated and tied with thread at the pyloric end and the ileocaecal junction. Then the weight of filled intestine was measured and the content was drained into a graduated cylinder and volume was measured, later the weight of empty intestine was re-measured again and the change in the full and empty intestine was calculated. Finally, the percentage reduction of intestinal discharge (volume) and weight of intestinal content were calculated by comparing with a negative control by the following formula [10, 12].

$$ \mathrm{Mean}\ \mathrm{Percentage}\ \mathrm{volume}\ \mathrm{inhibition}=\frac{\mathrm{MVICC}-\mathrm{MVICT}\ }{\mathrm{MVICC}} \times 100 $$

Where, MVICC is the Mean volume of the intestinal content of the control group

MVICT is the Mean volume of the intestinal content of the test group.

$$ \mathrm{Mean}\ \mathrm{Percentage}\ \mathrm{weight}\ \mathrm{inhibition}=\frac{\mathrm{MWICC}-\mathrm{MWICT}\ }{\mathrm{MWICC}}\times 100 $$

Where, MWICC is the Mean weight of the intestinal content of the control group

MWICT is the Mean weight of the intestinal content of the test group.

#### In vivo anti-Diarrhoeal index

The in vivo anti-diarrhoeal index (ADI) was determined by combining three parameters taken from the above-mentioned models. It was then expressed according to the following formula developed by Aye-than et al., [33].

$$ \mathrm{ADI}=\sqrt[3]{Dfreq\ X\  Gmeq\ X\  Pfreq} $$

Where, Dfreq is the delay in defecation time as a percentage of negative control,

Gmeq is the gut meal travel reduction as a percentage of negative control.

Pfreq is the reduction in purging frequency in the number of wet stools as a percentage of the negative control.

$$ \mathrm{Dfreq}=\frac{\mathrm{MODTG}-\mathrm{MODCG}}{\mathrm{MODCG}} $$

Where, MODTG is Mean onset of diarrhoea in the test group

MODCG is Mean onset of diarrhoea in the control group

### Antibacterial activity test

#### Media preparation and inoculum standardization

The standard bacterial medium was prepared and used according to the manufacturers’ guidelines. According to the Clinical and Laboratory Standard Institute (CLSI), the bacterial turbidity of each species was prepared and standardized. The turbidity of the inoculum tube was adjusted visually by the naked eye against a 0.5 McFarland turbidity equivalence with white background and contrasting black lines in the presence of adequate light by either adding colonies or sterile normal saline solution. It is assumed to contain a bacterial concentration of 1 × 10^8^ CFU/mL. Then, the standardized suspension was used within 15 min of its preparation [34].

#### Agar well diffusion method

The antibacterial activity of the crude root extract of *B. antidysenterica* was done according to Umer et al., and Molla et al., [32, 35]. It was performed on both American type cell culture (ATCC) and clinical isolates of selected intestinal pathogens obtained from Ayder comprehensive specialized hospital, which were considered as a major cause of diarrhoea such as *Shigella flexneri* (*S. flexneri*) (ATCC-12022), *Salmonella typhi* (*S. typhi*) (ATCC-13062), *Salmonella* species (clinical isolate), *E. coli* (ATCC-25922), *E. coli* (Clinical isolate) and *P. aeruginosa* (ATCC-27853).

Three test concentrations of plant extract (800 mg/mL, 400 mg/mL and 200 mg/mL), vehicle and standard antibiotic disc were used for determining zone of inhibition. Ceftazidime (30 μg/ disc, for *P. aeruginosa*) and ciprofloxacin (0.005 mg/disc, for the remaining bacteria) were used as a standard drug and distilled water was used as a negative control. The standard antibacterial discs were selected based on the susceptibility of bacterial species [36]. The antibacterial activity was evaluated by measuring the zone of inhibition against the test organisms by using vernier calliper and then compared with the reference as well as the control. Each procedure was done in triplicates and average values were being taken for further use [32, 35].

#### Determination of the minimum inhibitory concentration

Microdilution broth method was used for determining minimum inhibitory concentration (MIC) [21, 35]. MIC was performed for the test extract that showed acceptable (> 7 mm) antibacterial activity in agar well diffusion assay [21]. The plant extract concentration was ranged from 50 mg/mL to 0.0975 mg/mL for *S. flexneri* and from 200 to 0.39 mg/mL for the remaining bacteria in a descending order from first to tenth column.

The 96 well plates were used for resazurin-based microtitre dilution assay. The lowest concentration of test extract at which no colour change occurred was recorded as the MIC value [21, 35]. Each procedure was carried out in triplicates and average values were taken. In this experimentation, each microtiter plate had a set of two controls. One of the controls was growth control (colour contrast control) which contained all solution except the plant extracts (11th column). It was used to determine the growth of bacteria. The other control was sterility control which was the 12th column contained media and resazurin solution. The sterility control was used to know the sterility of the procedure (the media and resazurin solution). Finally results were compared with reference drug in the CLSI guideline and other literatures [34].

#### Determination of minimum bactericidal concentration

The minimum bactericidal concentration (MBC) was determined by sub-culturing the contents of wells from the MIC results for individual bacterium as done by previous literature [21, 35]. In this method, the contents of all wells resulting in MIC and all concentrations above MIC were streaked using a sterile wire loop on MHA. Then it was incubated at 37 °C for 24 h. The lowest concentration of the extract which showed no bacterial growth was distinguished and verified as the MBC. All procedures were conducted in triplicates and the average value was taken for the MBC of test material against each bacterium.

### Preliminary phytochemical screening

The qualitative phytochemical screening for the crude root extract of *B. antidysenterica* were done by using standard chemical tests [12, 37].

### Statistical analysis

Results were expressed in mean ± standard error of the mean (SEM) and comparisons were made between negative control, positive control and treatment groups of various doses using one-way analysis of variance (ANOVA) followed by *posthoc* Tukey’s test using statistical package for the social sciences (SPSS) version 20.0. All data were analyzed at 95% confidence interval and *P*-values less than 0.05 was considered as statistically significant. Coefficient of determination (linear regression analysis) (R^2^), was also determined by using Microsoft excel 2013. The analyzed data were presented using tables and figures.

## Results

### Acute Oral toxicity test

After a single oral administration of limit dose (2000 mg/kg), animals did not show any change in motor activity, lacrimation, diarrhoea, convulsion, and coma. In addition, no physical, behavioural and neurological changes were recorded. During the total period of acute toxicity study, none of the animals show any change in weight and appetite (food and water intake). No mortality was also recorded during the total 14 days of the observation period.

### Anti-diarrhoeal activity

#### Effects on Castor oil induced Diarrhoea in mice

The crude root extract of *B. antidysenterica* showed a dose-dependent response in prolonging the onset of diarrhoea. As shown in Table 1**,** the onset of diarrhoea was significantly protracted at doses of 200 and 400 mg/kg as compared to the negative control (*p* < 0.001). In addition, the largest test dose has shown maximum prolongation in diarrhoeal onset as compared to the standard drug loperamide 3 mg/kg. Moreover, all test doses of the crude root extract significantly decreased (*p* < 0.001) the average number and weight of wet faces as compared to the negative control.

**Table 1:** The Antidiarrhoeal effects of the root extract of *B. antidysenterica* on castor oil-induced diarrhoeal model in mice

	Dose Administered	Onset of Diarrhoea (Min)	No of wet faeces	Total No of faeces	Average weight of wet faeces (gm)	Average weight of total faeces (gm)	% Reduction
**80% BAE**	DW 10 mL/kg	76.17 ± 2.07	7.5 ± 0.43	8.17 ± 0.48	0.76 ± 0.04	0.83 ± 0.05	–
	Loperamide 3 mg/kg	166.33 ± 6.97^a3c3^	1.33 ± 0.33^a3c3^	2.67 ± 0.42^a3c2^	0.14 ± 0.03^a3c2^	0.23 ± 0.05^a3c2^	82.22%
	BAE 100 mg/kg	89.50 ± 2.50^b3d3e3^	3.5 ± 0.22^a3b3d3e3^	4.83 ± 0.31^a3b2e2^	0.38 ± 0.04 ^a3b2d1e3^	0.50 ± 0.05^a3b2e2^	53.33%
	BAE 200 mg/kg	151.00 ± 3.17^a3c3e2^	1.33 ± 0.21^a3c3^	3.33 ± 0.33^a3^	0.18 ± 0.03 ^a3c1^	0.31 ± 0.04^a3^	82.22%
	BAE 400 mg/kg	172.67 ± 3.48^a3c3d2^	0.83 ± 0.31^a3c3^	2.17 ± 0.40^a3c2^	0.11 ± 0.04 ^a3c3^	0.19 ± 0.05^a3c2^	88.89%

Data’s are mean ± SEM (*n* = 6); ^a^ compared with negative control values; ^b^ compared with loperamide; ^c^ compared with 100 mg/kg; ^d^ compared with 200 mg/kg; ^e^ compared with 400 mg/kg; ^1^*p* < 0.05, ^2^*p* < 0.01, ^3^*p* < 0.001; *DW* Distilled water, *BAE B. antidysenterica* extract

**Fig. 1:**
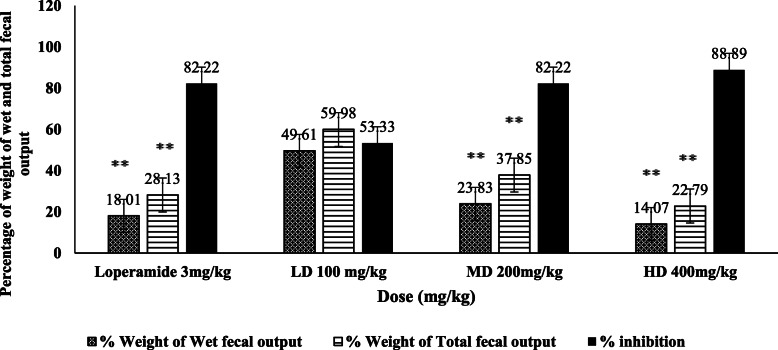
Percentage weight faecal output inhibition of the root extract of *B. antidysenterica* in castor oil-induced model. LD, low dose; MD, middle dose; HD, high dose; ** *p* < 0.001

As presented in Fig. 1***,*** the dose-dependent decrement in percentage weight of wet faecal output and percentage weight of the total number of faecal outputs were recorded. The largest dose showed the highest inhibition of defecation (88.89%), the lowest percentage of mean wet faecal output (14.07%) and a total number of faecal output (22.79%) as compared with all tested doses of the extract and positive control (*p* < 0.001).

#### Effects on Castor oil induced intestinal transit in mice

Results from the charcoal meal intestinal transit model revealed that the crude extract of *B. antidysenterica* showed significant inhibition of intestinal transit at all test doses. The maximum antimotility effect was recorded in the highest dose of test extract 400 mg/kg (67.59%), which is comparable with the standard drug loperamide 3 mg/kg (67.35%, *p* < 0.001) (Table 2).

**Table 2:** The effects of root extract of *B. antidysenterica* on gastrointestinal transit in mice

Extract	Dose Administered	Length of small intestine (cm)	Distance covered by charcoal meal (cm)	Peristaltic index (%)	% Inhibition
80% BAE	DW 10 mL/kg	57.50 ± 0.43	50.33 ± 0.84	87.56 ± 1.61	–
	Loperamide 3 mg/kg	57.83 ± 0.83	16.50 ± 0.76^a3c3d3^	28.59 ± 1.50 ^a3c3d3^	67.35
	BAE 100 mg/kg	57.83 ± 0.79	30.83 ± 0.67^a3b3d3e3^	53.30 ± 0.67 ^a3b3d3e3^	39.13
	BAE 200 mg/kg	58.33 ± 0.54	22.83 ± 0.84^a3b3c3e3^	39.11 ± 1.18 ^a3b3c3e3^	55.34
	BAE 400 mg/kg	56.83 ± 0.48	16.17 ± 1.19^a3c3d3^	28.38 ± 1.87 ^a3c3d3^	67.59

Data’s are mean ± SEM (*n* = 6); ^a^ compared with negative control values; ^b^ compared with loperamide; ^c^ compared with 100 mg/kg; ^d^ compared with 200 mg/kg; ^e^ compared with 400 mg/kg; ^1^*p* < 0.05, ^2^*p* < 0.01, ^3^*p* < 0.001; *DW* distilled water, *BAE B. antidysenterica* extract

#### Effects on Castor oil induced Enteropooling

The 80% hydro-methanolic crude root extract of *B. antidysenterica* showed a significant reduction in both average weight and volume of intestinal contents as compared with the negative control. Significant inhibition of intestinal milked volume was recorded on 200 mg/kg (*p* < 0.05) and 400 mg/kg (*p* < 0.01) test dose relative to the negative control group. The highest dose of test extract 400 mg/kg showed comparable result (37.76%, *p* < 0.01) as of standard drug Loperamide 3 mg/kg (39.80%, *p* < 0.01) (Table 3).

**Table 3:** The effects of the root extract of *B. antidysenterica* on gastrointestinal fluid accumulation in mice

Extract	Dose Administered	Volume of intestinal contents (mL)	% Inhibition	Weight of intestinal contents (gm)	% Inhibition
80% BAE	DW 10 mL/kg	0.82 ± 0.03	–	1.07 ± 0.04	–
	Loperamide 3 mg/kg	0.49 ± 0.04^a3c2^	39.80	0.71 ± 0.03^a2^	34.31
	BAE 100 mg/kg	0.71 ± 0.03^b2e2^	13.27	0.90 ± 0.09	16.25
	BAE 200 mg/kg	0.57 ± 0.05^a3^	30.61	0.78 ± 0.06^a1^	27.82
	BAE 400 mg/kg	0.51 ± 0.02^a3c2^	37.76	0.72 ± 0.06 ^a2^	32.56

Data’s are mean ± SEM (*n* = 6); ^a^ compared with negative control values; ^b^ compared with loperamide; ^c^ compared with 100 mg/kg; ^d^ compared with 200 mg/kg; ^e^ compared with 400 mg/kg; ^1^*p* < 0.05, ^2^*p* < 0.01, ^3^*p* < 0.001; *DW* distilled water, *BAE B. antidysenterica* extract

The other parameter recorded in this model was percentage inhibition in the average weight of intestinal contents. As shown in Table 3, significant inhibition were recorded in 200 and 400 mg/kg dose of test extract with percentage inhibition of 27.82% (*p* < 0.05) and 32.56% (*p* < 0.01) respectively. The highest level of intestinal fluid reduction was recorded in the standard drug loperamide 3 mg/kg (34.31%), which is comparable with the highest dose of test extract.

#### In vivo anti-diarrhoeal index

As shown in Table 4**,** 32.04, 73.22 and 87.6% were recorded as in vivo ADI for 100, 200 and 400 mg/kg dose of test extract. The 400 mg/kg dose showed the highest in vivo anti-diarrhoeal index, which is comparable to the standard drug loperamide 3 mg/kg dose (83.07%).

**Table 4:** In vivo antidiarrhoeal index of the root extract of *B. antidysenterica*

Extract	Dose Administered	Delay in defecation time (Dfreq) (%)	Gut meal travel distance (Gmeq) (%)	Purging frequency in number of wet stools (%)	In vivo Anti-diarrhoeal Index (ADI)
80% BAE	DW 10 mL/kg	–	–	–	–
	Loperamide 3 mg/kg	118.37	67.35	82.22	83.07
	BAE 100 mg/kg	17.50	39.13	53.33	32.04
	BAE 200 mg/kg	98.24	55.34	82.22	73.22
	BAE 400 mg/kg	126.69	67.59	88.89	87.60

*BAE B. antidysenterica* extract, *DW* distilled water

### Antibacterial activity

#### Antibacterial zone of inhibition

The concentration-dependent effect (R^2^) values were calculated for each bacterial species accordingly, R^2^ of 80% methanolic extract was 0.873, 0.890, 0.930 and 0.987, for ATCC strain of *S. typhi, E. coli, P. aeruginosa* and *S. flexneri* respectively. The maximum average zone of inhibition was recorded at 800 mg/mL concentration of test extract as 22.0, 20.7, 19.3 and 18.3 mm for ATCC strains of *P. aeruginosa*, *S. flexneri*, *S. typhi* and *E. coli* respectively. On the contrary, no bacterial growth inhibition was observed in all concentrations of 80% methanolic extract against clinical isolates of *E. coli* and *Salmonella* species.

In Table 5, the mean comparison of different concentrations of extract within a group and with the standard antibacterial disc was presented. As shown in the table, all concentrations of plant extract showed a statistically significant difference (*p* < 0.001) as compared with the positive control in all standard bacteria. In addition, the 200 mg/mL concentration of extract had significant difference (*p* < 0.01) for *S. typhi* and *E. coli* and *p* < 0.001 for *P. aeruginosa* as compared with 400 mg/mL of crude extract. It had also a *p*-value < 0.001 as compared with 800 mg/mL concentration for *S. typhi*, *E. coli* and *P. aeruginosa* while, the p-value is less than 0.01 for *S. flexneri*. In addition, the 400 mg/kg concentration of test extract showed a significant difference (*p* < 0.01) as compared with 800 mg/mL for *E. coli* and *P. aeruginosa*, while *p* < 0.05 for *S. flexneri*.

**Table 5:** Zone of inhibition for the root extract of *B. antidysenterica* against selected diarrhoea causing bacteria

Extract	Concentration	*S. flexneri* (ATCC-12022) (mm)	*S. typhi* (ATCC-13062) (mm)	*Salmonella Spp.* (CI) (mm)	*E. coli* (ATCC-25922) (mm)	*E. coli* (CI) (mm)	*P. aeruginosa* (ATCC-27853) (mm)
80% BAE	BAE 200 mg/mL	14.7 ± 0.33 ^a3d2^	14.7 ± 0.33 ^a3c2d3^	NA	14.0 ± 0.00 ^a3c2d3^	NA	16.7 ± 0.33 ^a3c3d3^
	BAE 400 mg/mL	17.3 ± 0.67 ^a3d1^	17.7 ± 0.33 ^a3b2^	NA	16.7 ± 0.33 ^a3b1d2^	NA	19.7 ± 0.33 ^a3b3d2^
	BAE 800 mg/mL	20.7 ± 0.33 ^a3b2c1^	19.3 ± 0.33 ^a3b3^	NA	18.3 ± 0.33 ^a3b1c3^	NA	22.0 ± 0.00 ^a3b3c2^
Standards	Cipro *5 μg*/disc	29.3 ± 1.20	34.3 ± 0.67	18.0 ± 0.00	32.3 ± 0.33	NA	–
	Cefta 30 μg/disc	–	–	–	–	–	10.3 ± 0.33

Data are expressed as Mean ± SEM (*n* = 3), ^a^ compared to positive control; ^b^ compared to 200 mg/mL; ^c^ compared to 400 mg/mL; ^d^ compared to 800 mg/mL; ^1^*P* < 0.05, ^2^*P* <0.01, ^3^*P* <0.001. The negative control has shown no antibacterial activity. The positive controls: *BAE Brucea antidysenterica* extract, *Cipro* Ciprofloxacin, *Cefta* Ceftazidime, *NA* No Activity

#### Minimum inhibitory concentration and minimum bactericidal concentration of crude extract against bacterial species

According to the result obtained from 96 well microtiter plate microdilution test, the minimum inhibitory concentration of the crude extract against the tested species were less than or equal to (≤) 3.13 mg/mL for all tested bacteria species. As shown in Table 6, the lowest MIC of the crude root extract was recorded as 0.39 mg/mL for *S. flexneri* and the highest is for *P. aeruginosa* with a concentration of 3.13 mg/mL.

**Table 6:** Minimum inhibitory concentration and minimum bactericidal concentration of 80% methanolic root extract of *B. antidysenterica*

Bacterial species	80% methanolic BAE		
	MIC (mg/mL)	MBC (mg/mL)	MBC/MIC
*S. flexneri*	0.39 ± 0.00	1.56 ± 0.00	4.00
*S. typhi*	1.56 ± 0.00	3.13 ± 0.00	2.00
*E. coli*	2.08 ± 0.52	8.33 ± 2.08	4.00
*P. aeruginosa*	3.13 ± 0.00	200 ± 0.00	63.89

Data are expressed as Mean ± SEM (*n* = 3); *BAE B. antidysenterica* extract

As presented in Table 6, the MBC values ranged from 1.56 mg/mL (*S. flexneri*) to 200 mg/mL (*P. aeruginosa*).

#### Bacteriostatic/ bactericidal/ nature of the crude root extract of *B. antidysenterica*

The bacteriostatic and bactericidal nature of the plant extract was also evaluated to assess its potential against bacterial strains. There are certain circumstances where cidal agents are preferable over static agents and vice versa [38]. Antibacterial substance is said to be bactericidal if the ratio of MBC/MIC is ≤4 and bacteriostatic if MBC/MIC > 4 [39]. As presented in Table 6, except *P. aeruginosa*, the crude plant extract showed bactericidal activity. Moreover, extracts with MICs values between 0.1 and 0.625 mg/mL had moderate activity [40].

## Phytochemical constituents

As presented in Table 7, the phytochemical analysis of the crude root extract of *B. antidysenterica* indicates the presence of different bioactive components.

**Table 7:** Phytochemical composition of the crude root extract of *B. antidysenterica*

Metabolites Tested	80% methanolic BAE
Alkaloids	+
Saponins	+
Tannins	+
Polyphenols	+
Terpenoids	+
Flavonoids	+
Anthroquinones	–

*BAE B. antidysenterica* extract, +: Present, −: Absent

## Discussion

Diarrhoea is characterized by rapid and frequent passage of semisolid or liquid faecal material. It involves decreased in absorption of fluid, increased motility of the intestinal tract and increased secretions. In addition, diarrhoea leads to loss of electrolytes particularly sodium (Na^+^) and water, and finally end up with dehydration and death [7, 41, 42].

80% methanol was used as a macerating solvent for plant extraction and it was similar with other laboratory works done on *B. antidysenterica* [20, 21]. In general, hydro-alcoholic co-solvents such as 80% methanol seem to possess the optimum solubility characteristics for initial crude extraction and provide high extraction yield [10, 43]. However, despite the several works interests in the polyphenols extraction, there is no single solvent which may be considered standard because it is usually different for different plant matrices [43].

Castor oil-induced diarrhoeal model is the first model to assess the ability of test extract towards its antidiarrhoeal activity [12]. It causes an imbalance between the secretary and absorptive processes in the small intestine by producing inflammation and local irritation [10, 12, 44]. After oral administration, “ricinoleic acid” the active constituent of CO, is released by intestinal lipases. Consequently, it activates intestinal smooth-muscle cells via endothelial prostanoid receptors (EP_3_). Then it induces fluid and electrolyte secretion secondary to stimulation of an active anion secretory process, which is mediated by cAMP [45–47]. Therefore, the use of castor oil as diarrhoea inducer for all models is reasonable since it resembles the pathophysiologic processes and ensures the actual diarrhoeal (secretory and inflammatory diarrhoea) diseases in humans.

Results in this experiment verified that the crude root extract of *B. antidysenterica* showed a dose-dependent response by prolonging the onset of diarrhoea on mice. The highest activity was seen in the two higher doses of test extract while the lower dose was devoid of significant prolongation. This might be associated with the smallest dose of test extract has not sufficient ability to prolong the onset of diarrhoea. As explained by other reports doses having lower antimotility and/or antisecretory effects are less likely to address all the parameters [10, 12, 15].

The root extract of *B. antidysenterica* also significantly decrease the average number and weight of wet faces in the castor oil-induced diarrhoea model. Studies proved that an increase in stool frequency and volume showed the severity of diarrhoea and also a decreased in wet faces was used as a sign of recovery [48]. According to Degu et al, a decrease in stool frequency was associated with antisecretory and antimotility mechanisms, by which it decreases the number and weight of wet stool [12]. In addition, a study done by Lakshmanan et al, stated that the decreased in stool volume was mediated either by an antisecretory mechanism, reduction in intestinal muscle tone and/or decreased peristalsis of the gastrointestinal tract, which can slow the movement of faecal matter through the GI tract [49].

Gastrointestinal motility was significantly decreased at all test doses of crude extract. Different studies confirmed that the peristalsis in the GI system was increased in case of diarrhoea [3, 50]. The reduction of GI peristalsis is one of the mechanisms by which antidiarrhoeal agents can act. For example, the standard drug (loperamide) used in this study acts by activation of μ receptors that inhibit the release of acetylcholine to enhance phasic colonic segmentation and inhibit peristalsis, thus increasing intestinal transit time [51]. The report was compared with similar reports done by Degu et al and Sisay et al [10, 12]. All the test doses of plant extracts showed a significant decrement in peristaltic movement.

The third model in this study was castor oil-induced enteropooling model, which is aimed to assess the potential inhibition of secretory components in the gastrointestinal tract after castor oil administration for the induction of diarrhoea. Outcomes in this model proved that the 80% methanolic extract of *B. antidysenterica* showed a dose-dependent reduction in both average weight and volume of intestinal contents at all test doses as compared with the negative control. Even though the maximum effect was seen in the standard drug, the highest dose of plant extract exhibit a comparable effect. As described in Beubler, the gastrointestinal secretions secondary to castor oil administration are related to ricinoleic acid, which activates the nitric oxide pathway and induces nitric oxide (NO) dependent gut secretion along with prostaglandin synthesis. So that the possible mechanism is illustrated as inhibition of the nitric oxide pathway, by that it halts GI secretion [52].

The combined effect of antidiarrhoeal agents was generally investigated by calculating ADI [33]. As presented in Table 4, ADI values showed the dose-dependent nature of each parameter. The highest dose of the crude extract showed highest ADI as compared with corresponding doses and also better than the standard drug. This might be due to its better potential in prolongation of onset of diarrhoea, decrement of peristaltic movement and halting of purging frequency in the GI system as compared with the standard drug and respective doses.

Different mechanisms are hypothesized as a mechanism for the antidiarrhoeal effect of *B. antidysenterica*. Compounds inhibiting prostaglandin inhibition has an ability to prevent diarrhoea [53]. Similarly, a study done by Tessema et al, showed that *B. antidysenterica* has an anti-inflammatory effect, which might be attributed to inhibition of castor oil-induced prostaglandin synthesis and prevention of diarrhoea by inhibiting stimulation of intestinal secretions [54].

The other activity done in this study was the determination of antibacterial activity for selected diarrhoea causing bacteria. Based on the results obtained the highest antibacterial activity was observed in the highest concentration (800 mg/mL) of crude extract. The maximum zone of inhibition was recorded for ATCC strain of *P. aeruginosa.* Although the test extract has a smaller zone of inhibition as compared with the positive control in the case of *S. flexneri, S. typhi* and *E. coli,* it has better zone of inhibition for *P. aeruginosa*. This finding was inline with a study done on the leaf extract of *B. antidysenterica* [22]*.* According to Fentahun et al, the chloroform leaf extract of *B. antidysenterica* has showed highest (11 mm) zone of inhibition [22]. Other results done on different plants against *P. aeruginosa* showed that the zone of inhibition was ranging between 9 and 21 mm [23, 32, 35, 55, 56]. This may indicate that the root extract of *B. antidysenterica* has been promising activity against *P. aeruginosa* and important to tackle diarrhoea secondary to chronic diseases.

The MIC and MBC results from this experiment showed that the root extract of *B. antidysenterica* has the ability to inhibit and kill bacteria. The MIC of an extract is regarded as good if the values are less than 0.1 mg/mL, moderate if it is between 0.1 and 0.625 mg/mL and weak when it is above 0.625 mg/mL [40, 57]. Based on this, the MIC of 80% hydro-methanolic crude extract of *B. antidysenterica* was ranged from moderate to weak against the tested bacteria strains. This might be related with their zone of inhibition during agar well diffusion assay. According to CLSI (2015a), compounds having a higher zone of inhibition has an ability to inhibit bacterial growth with a smaller concentration as compared with compounds having a low zone of inhibition. In addition, MBC of plant extract were one or more dilution factor greater than MIC values for each bacteria except for *P. aeruginosa*. It may indicate the sensitivity of test extract for those common diarrhoea causing bacteria [21]. However, *P. aeruginosa* is known to utilize its high level intrinsic and acquired resistance mechanisms to counter most antibiotics [58]. In addition, recent finding showed that biofilm-mediated resistance and formation of multidrug-tolerant persister cells are responsible for recalcitrance and relapse of infection [59]. On the other hand, the promising activity of plant extract against *S. flexneri, S. typhi and E. coli* might be used for treatment of bloody diarrhoea [60], inflammatory induced diarrhoea [61], traveller diarrhoea and food poisoning [62] respectively.

Results of phytochemical screening also support some of the aforementioned mechanisms listed above. According to similar studies done on different plants suggested that the presence of tannins and flavonoids increase colonic water and electrolyte reabsorption. Tannins are known for making intestinal mucosa more resistant by reducing secretion, normalizing deranged water transport and reduction of intestinal transit [12, 63]. Other secondary metabolites like terpenoids and saponins also have the ability to inhibit the release of autacoids like prostaglandins and histamines [16]. Phytochemicals such as phenolic compounds and alkaloids also inhibit intestinal motility [13, 63, 64]. In addition, flavonoids are also found to display a wide range of biological activities including inhibition of enzymes such as prostaglandin synthase, cyclooxygenase and lipoxygenase that might mainly contribute to its anti-diarrhoeal activity [65].

The bioactive components, especially tannins and flavonoids, exert a major role on antibacterial activity. Different mechanisms are postulated for the action of these bioactive components. For example, tannins produce antibacterial activity by inactivating numerous enzymes, microbial adhesion, and cell envelope transport proteins [35]. Other active components like saponins have been reported to possess antibacterial activity, which could be attributed to their ability to form a complex with extracellular proteins, soluble proteins, and bacterial cell walls [55]. Other previous antibacterial studies also proved that the presence of these secondary metabolites has an important role in the inhibition of bacterial growth [21, 54, 55].

## Conclusion

Based on this study, the 80% hydro-methanolic root extract of *B. antidysenterica* had promising anti-diarrhoeal and antibacterial activities. The overall antidiarrhoeal activity of the studied plant extract was associated with inhibitory effects on castor oil-induced gastrointestinal motility and fluid secretion. In addition, it has bactericidal activity against most of the studied bacteria. Further studies for solvent fractions, isolation of active principle (s), chemical standardization and elucidating the possible mechanism of action are recommended.

## Data Availability

The datasets generated and/or analyzed during the study are available from the corresponding author on reasonable request.

## Abbreviations

ATCC: American type of cell culture
CFU: Colony-forming unit
CLSI: Clinical and laboratory standard institute
MBC: Minimum bactericidal concentration
MIC: Minimum inhibitory concentration
SPSS: Statistical package for the social sciences
